# Awareness of Voting Rights Among Psychiatric Inpatients – Patients Should Affect Policies

**DOI:** 10.1192/bjo.2025.10103

**Published:** 2025-06-20

**Authors:** Rakesh Puli, Dawood Razzak, Olaide Oladosu, Nicolas Upton, Katie Fergus

**Affiliations:** Cardiff and Vale, Cardiff, United Kingdom

## Abstract

**Aims:** Mental health inpatients are eligible to vote, whether they are detained under the Mental Health Act or not (except for those under a forensic section). It is essential that we do everything possible to facilitate patients being able to vote. Psychiatric inpatients not exercising their democratic right due to logistical failings would further implicate our mental health services in the systemic stigma that people with mental health difficulties face.

1) Assess patient’s knowledge of their voting rights

2) Assess proportion of the inpatients currently registered to vote

3) Explore any misinformation patients have been given in the past with regards to their voting rights

4) Explore patient’s willingness to advocate for future mental health policy changes as someone with lived experiences of mental health services.

**Methods:** Type of patients: Functional psychiatric inpatients who are both informal and under section in Cardiff and Vale University health board. Data time frame: 25/06/2024 to 04/07/2024 (prior to 2024 general election in UK). We received 67 responses in total from 8 wards.

Method of collection: A proforma filled out by consenting patients in all the adult psychiatry inpatient wards and functional ward of old age Psychiatry in University Hospital Llandough, Wales and community inpatient wards under Rehabilitation Psychiatry in Cardiff.

**Results:** Awareness of voting rights of patients is low, among patients and staff alike, on informal interactions. 76% of the responders were aware of the upcoming election and 64% were aware of their voting rights (2024 general election). There was a mixed response in patients wanting to vote in the recent general election as only 55% shared their intention to vote. 10 patients (14%) reported being told by someone that they were ineligible to vote. 60% of the patients were aware of the need for a photo ID to vote and 53% had a photo ID. 33 patients (around 50%) expressed willingness to advocate for future changes to policies.

**Conclusion:** 1) While there is interest in inpatient settings to influence change in the political setting, more work must be done to educate and inform the inpatient population of their voting rights.

2) Work should be done to make the process of registration for voting streamlined for inpatients, including supporting their access to a photo ID.

3) Arrangements should be made to allow voting by the most suitable method (i.e. in person, by post or by proxy).

